# Leukocyte telomere length in patients with transfusion-dependent thalassemia

**DOI:** 10.1186/s12920-020-00734-9

**Published:** 2020-06-01

**Authors:** Nithita Nanthatanti, Adisak Tantiworawit, Pokpong Piriyakhuntorn, Thanawat Rattanathammethee, Sasinee Hantrakool, Chatree Chai-Adisaksopha, Ekarat Rattarittamrong, Lalita Norasetthada, Wirote Tuntiwechapikul, Kanda Fanhchaksai, Pimlak Charoenkwan, Sirinart Kumfu, Nipon Chattipakorn

**Affiliations:** 1 Division of Hematology, Department of Internal Medicine, Faculty of Medicine, Chiang Mai University, 110, Inthavarorose Rd, Suthep, Muang, Chiang Mai, 50200 Thailand; 2 Department of Biochemistry, Faculty of Medicine, Chiang Mai University, Chiang Mai, Thailand; 3 Division of Hematology and Oncology, Department of Pediatrics, Faculty of Medicine, Chiang Mai University, Chiang Mai, Thailand; 4 Cardiac Electrophysiology Research and Training Center (CERT Center), Department of Physiology, Faculty of Medicine, Chiang Mai University, Chiang Mai, Thailand

**Keywords:** Iron overload, Oxidative stress, TDT, Telomere, Transfusion dependent thalassemia

## Abstract

**Background:**

Thalassemia is a hereditary hemolytic anemia with a severity ranging from mild, non-transfusion dependent to severe chronic anemia requiring lifelong transfusion. Transfusional iron overload is a major complication in patients with transfusion-dependent thalassemia (TDT). Telomeres are sequences of nucleotides forming the end caps of chromosomes that act as a DNA repair system. Iron overload in thalassemia can cause increased oxidative stress which leads to cellular damage and senescence. This may result in telomere length shortening. The degree of telomere length shortening may reflect the severity of thalassemia.

**Methods:**

This research aimed to study the leukocyte telomere length in patients with TDT in comparison to non-thalassemic individuals and to identify the clinical and laboratory parameters that are associated with telomere length. We conducted a cross-sectional study in patients with TDT aged ≥18 years. Leukocyte telomere length was measured by real-time quantitative PCR.

**Results:**

Sixty-five patients with TDT were enrolled onto the study. There were 37 female patients (54.4%). The median age was 27 (18–57) years, and mean pre-transfusion hemoglobin level was 7.1 (± 1.07) g/dL. The mean telomere to single copy gene (T/S) ratios of patients with TDT and the controls were 0.72 ± 0.18 and 0.99 ± 0.25, respectively (*p* < 0.0001). There was a significant correlation between the T/S ratio and age (*p* = 0.0002), and hemoglobin level (*p* = 0.044). There was no correlation between telomere length and other factors.

**Conclusions:**

Our study showed that TDT patients had shorter leukocyte telomere length compared with controls. Leukocyte telomere shortening in TDT was an aging-dependent process and associated with lower hemoglobin level.

## Background

Thalassemia is a common hereditary hemolytic anemia in Thailand. About 30–40% of Thai population carries at least one of the abnormal globin genes [1]. Clinical severity of thalassemia varies greatly and could be classified as non-transfusion dependent thalassemia (NTDT) which requires occasional blood transfusion in specific situations and transfusion dependent thalassemia patients (TDT) which requires regular blood transfusion [2, 3]. Long-term blood transfusion and increased iron absorption from the gastrointestinal tract lead to iron overload, which can result in peroxidative damages to cells, organelles and membranes. The cellular damage associated with iron overload is mainly mediated by the state of oxidative stress and the effect of free oxygen radicals on various cell components and tissue [4–6].

Telomeres are the terminal section of chromosomes consisting of hexameric protein repeats amino acid (5′-TTAGGG-3′). The telomeres play roles in stabilization of chromosomes, protection of DNA from end to end fusion and reparation of DNA. At each cell division, telomeres become shorter and finally reach a critically short length when their function will be lost. This means that cells can no longer divide and results in cellular aging and apoptosis [7, 8].

The length of the telomeres in leukocytes can be used as an indication of good health, aging and longevity of the cell. Some human disorders associated with shorter telomere length originate from defective function of telomerase, an enzyme that extends the telomeres of chromosomes. Certain diseases originating from mutations in genes controlling the DNA repair system also result in accelerated telomere shortening and premature aging. One such condition is aplastic anemia. The mechanism of anabolic hormones is their effect on telomere length. The anabolic hormones are used to treat aplastic anemia and telomere-associated diseases. According to this information, development of the drugs that effect telomere length or factors that influence telomere length will be useful for thalassemia patients [7–10].

There is limited data about telomere length in patients with thalassemia. Chaichompoo et al reported that the rate of telomere shortening in patients with beta-thalassemia/hemoglobin E disease was accelerated when compared to normal individuals. Oxidative stress is one of the factors contributing to erosion of telomeres and cell replication capability. It was also demonstrated that the telomere shortening was associated with the clinical severity of thalassemia [11].

There is limited data about telomere length in TDT patients who have more iron overload and oxidative stress. We conducted the study in patients with TDT who had severe clinical symptoms. Both alpha and beta thalassemia patients were included to define the clinical factors that were associated with telomere shortening.

## Methods

### Study population and definition

We conducted a cross-sectional study in patients with TDT, aged 18 years and up, who attended Adult Hematology clinic at Chiang Mai University Hospital. TDT was defined as a patient with thalassemia who required red cell transfusion at least 3 times per year.

### Clinical and laboratory measurement

We collected clinical and laboratory parameters at the time of enrollment to identify factors associated with telomere length including age, sex, history of splenectomy, iron chelation, and factors associated with clinical severity including: pre-transfusion Hb level (mean steady-state Hb level in the previous 10 visits), units of red blood cell transfusion per month, serum ferritin (maximum, minimum, mean of serum ferritin at multiple times every year). All patients were investigated for telomere length, liver function test (LFT), non-transferrin bound iron (NTBI), hemolysis parameters, MRI for cardiac T2 star (T2*) and liver iron concentration (LIC). Thirty healthy individuals, age and sex-matched, were included as controls and were tested for telomere length.

### Blood samples and genomic DNA extraction

Blood samples (10 mL) were collected from the patients and healthy individuals in ethylenediaminetetraacetic acid (EDTA) tubes. Peripheral blood leukocytes were collected from blood samples by gradient density centrifugation through HiSep (HIMEDIA, Mumbai, India) and stored at − 80 °C before they were used. In our study, we used the Blood DNA Extraction Kit (OMEGA, Norcross, GA, USA) to extract genomic DNA from the peripheral blood leukocytes.

### Telomere length measurement

Telomere length was measured by using a real-time quantitative PCR as described by Cawthon, RM et al. [12]. The telomere/single copy gene (T/S) ratio was calculated as the ratio of telomere repeat copies (T) to single-copy gene copy number and albumin gene (S) to represent the average length of telomere, which by definition is 1.00. The specificity of all amplifications was determined by a melting curve analysis. DNA from healthy individuals which have a T/S ratio of 1.00 were used as a reference DNA sample (the ‘standard DNA’). Four concentrations of the standard human genomic DNA samples were prepared by 3-fold serial dilutions (70 ng, 23.33 ng, 7.77 ng, and 2.59 ng per well), and aliquoted in duplicate to a 96-well PCR plate. The primers for amplification of the scg albumin are albumin forward primer, albu: 5′-CGGCGGCGGGCGGCGCGGGCTGGGCGGAAATGCTGCACAGAATCCTTG-3′ and albumin reverse primer, albd: 5′-GCCCGGCCCGCCGCGCCCGTCCCGCCGGAAAAGCATGGTCGCCTGTT-3′; telomere forward primer, telg, 5′-ACACTAAGGTTTGGGTTTGGGTTTGGGTTTGGGTTAGTGT-3′ and telomere reverse primer, telc, 5′-TGTTAGGTATCCCTATCCCTATCCCTATCCCTATCCCTAACA-3′. The thermal cycling profile was Stage 1: 15 min at 95 °C; Stage 2: 2 cycles of 15 s at 94 °C, 15 s at 49 °C; and Stage 3: 32 cycles of 15 s at 94 °C, 10 s at 62 °C, 15 s at 74 °C with signal acquisition, 10 s at 84 °C, 15 s at 88 °C with signal acquisition. All samples were assayed in triplicates in order to minimize the sample-to-sample variation. Once PCR has completed, the Applied Biosystems QuantStudio™ 6 Flex Real-Time PCR analysis software was used to determine the T and S values for each experimental sample based on the standard curve method (Applied Biosystems, Foster City, CA, USA). The results of telomere length from PCR were expressed as T/S ratio.

### Statistical analysis

Mean telomere length (T/S ratio) of patients was compared with the ratio obtained from the controls by using a Student’s T-test. We examined the association of clinical factors and the T/S ratio using ANOVA (F-test). Multivariable regression analysis was performed using a forward selection technique. We proposed to examine age, pre-transfusion hemoglobin level and type of iron chelator. The correlation coefficient between the T/S ratio and each parameter value was calculated using Pearson’s correlation. The significance level was set at a *p* value < 0.05.

### Compliance with ethical standards

Informed consents were completed by all patients before enrollment. This study was approved by the Human Research Ethics Committee of Faculty of Medicine, Chiang Mai University. (study code: MED-2559-03967).

## Results

Sixty-five TDT patients were enrolled onto this study. There were 37 female patients (57.4%). The median age was 27.0 years (range 18–57). The mean pre-transfusion Hb level was 7.1 (± 1.07) g/dL. The beta-thalassemia/Hb E was the majority with 39 patients (57.4%) as shown in Table 1. Thirty age and sex-matched healthy subjects were enrolled in the control group. The median age was 30 (18–50) years. There were 19 females (63.33%). The age and gender distribution were not different between the patient and control groups (*p* = 0.07 and *p* = 0.55 respectively).

**Table 1:** The clinical characteristics of enrolled patients

Clinical characteristics	*N* = 65 (%)
Female	37 (54.4)
Median age (year) (range)	27.0 (18–57)
Baseline pre-transfusion Hb level (g/dL) (range)	7.1 (4.8–10.1)
Red blood cell transfusion (unit per month) (range)	1.67 (0.33–2)
Type of thalassemia	
Beta-thalassemia/Hb E disease	39 (57.4)
Homozygous beta-thalassemia	24 (35.3)
Hb H with Constant Spring disease	2 (2.9)
Splenectomy	46 (67.6)
Median serum ferritin level (mcg/dL) (range)	1499 (272–7371)
Median reticulocyte count (%) (range)	5.2 (0.27–31.2)
Median absolute reticulocyte count (×10^6^/mm^3^) (range)	155.4 (11–1370)
Median Cardiac T2* (ms)	38.3
> 20	54 (79.4)
10–20	2 (2.9)
< 10	5 (7.4)
Median LIC (mg/g of dry weight)	14.8 (1.3–27.0)
< 7	7 (11.7)
7–15	23 (38.3)
> 15	30 (50.0)
Liver enzymes (U/L)	
Median AST (range)	39.48 (16–136)
Median ALT (range)	34.98 (4–175)
Mean NTBI (micromol/L) (range)	6.94 (2.22–10.00)

AST Aspartate transaminase; ALT Alanine transaminase; LIC liver iron concentration;

NTBI Non-transferrin bound iron

### T/S ratio in TDT patients compared to control group

**Fig. 1:**
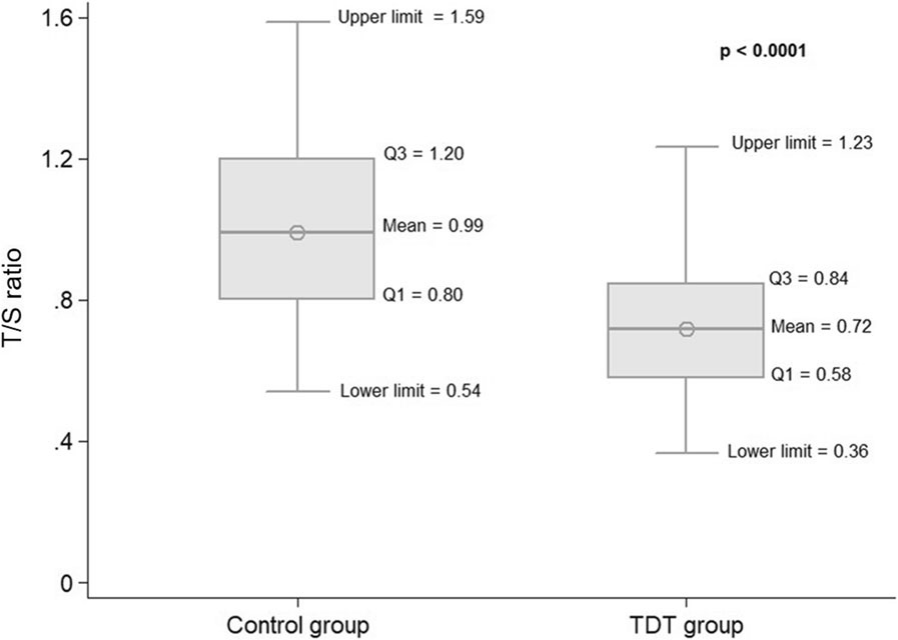
The T/S ratio of patients with TDT compared with controls

The mean T/S ratio of the TDT group was 0.72 ± 0.18. The mean T/S ratio of the control group was 0.99 ± 0.25. TDT patients had a statistically significant shorter T/S ratio compared to the control group (*p* < 0.0001). (Fig. 1).

### Factor associated with telomere length in TDT patients

**Fig. 2:**
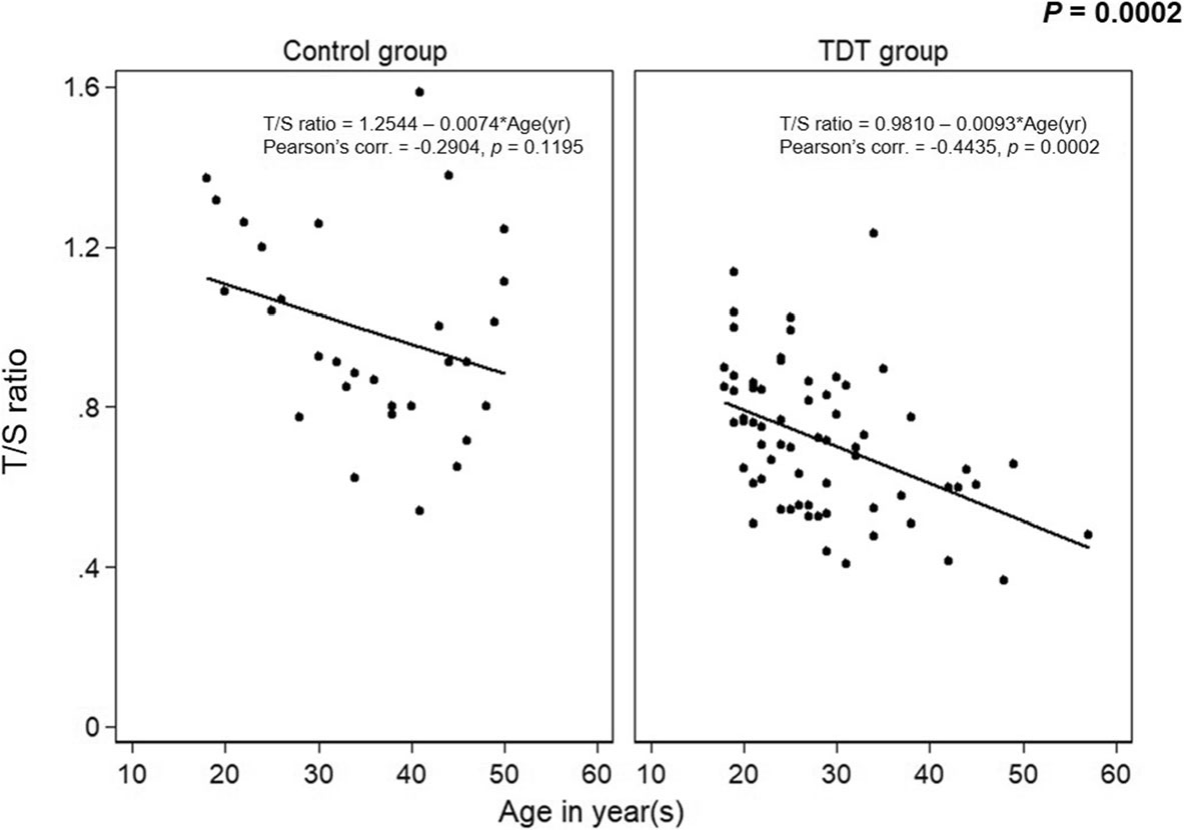
Correlation between T/S ratio and age in patients with TDT patients and controls

**Fig. 3:**
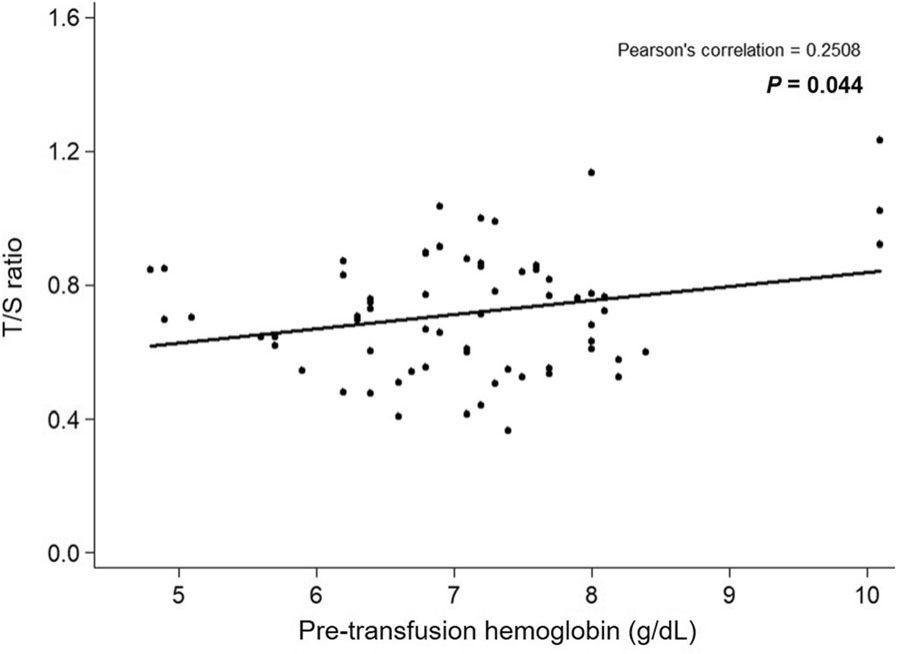
Pearson’s correlation between T/S ratio and pre-transfusion Hb level in patients with TDT

The correlation coefficients were calculated by Pearson’s method. Moderate negative correlations were seen between the mean T/S ratio and age (r = − 0.4435, *p* = 0.0001), Fig. 2, and there was a positive correlation with pre-transfusion Hb level (r = 0.2508, *p* = 0.044), Fig. 3.

The multivariate analysis factor showed that telomere length shortening was associated with age (*p* < 0.0001) and pre-transfusion Hb level (*p* = 0.02).

Sixty-three patients received iron chelation therapy, thirteen patients received deferoxamine monotherapy and 6 patients received deferoxamine combined with other iron chelator. Patients who received combination therapy had higher serum ferritin level more than patients who received monotherapy. There were 2 patients who did not receive iron chelation because of severe adverse effects from iron chelators. We found that the type of iron chelation therapy resulted in a difference in mean T/S ratio. Two patients who did not receive iron chelation had a shorter T/S ratio (0.41 and 0.60) compared to patients who received iron chelation, and patients who received deferoxamine as an iron chelator had the longest mean T/S ratio compared to other iron chelators (0.87) (*p* = 0.008).

There was no correlation between T/S ratio and other laboratory parameters including reticulocyte count, ferritin level, cardiac T2*, liver iron concentration, NTBI, or hemolysis parameters such as reticulocytes and liver enzymes. (Table 2) The multivariate analysis showed that only the age (*p* < 0.0001) and pre-transfusion Hb level (*p* = 0.027) were significantly associated with T/S ratio.

**Table 2:** Correlation coefficients and *p* values between T/S ratio and parameters in patients with TDT

Parameters	Correlation coefficient	*p* value
Age	−0.444	< 0.0001*
Rate of red blood cell transfusion	0.014	0.911
Pre-transfusion Hb level	0.251	0.044*
Maximum ferritin level	0.172	0.170
Reticulocyte count	0.215	0.105
CardiacT2*	−0.140	0.282
LIC	−0.020	0.879
Liver enzymes		
AST	−0.085	0.499
ALT	0.013	0.918
NTBI	0.216	0.120

Correlation coefficients were calculated by Pearson’s method

AST, Aspartate transaminase; ALT, Alanine transaminase; LIC, liver iron concentration;

NTBI, Non-transferrin bound iron; TDT, transfusion-dependent thalassemia

*Denotes statistical significance at *p* < 0.05

No difference was found between T/S ratio and gender (*p* = 0.068), type of thalassemia (*p* = 0.87), and splenectomy (*p* = 0.36). The majority of the population of patients had a high LIC [LIC 7–15 mg/g 25 (36.8%), > 15 mg/g 28 (41.2%)] and there was a trend of a lower T/S ratio in the high LIC group but this was not statistically significant (*p* = 0.6).

## Discussion

Telomere length is a well-known biomarker of cellular senescence [13–15]. Our study demonstrated that patients with TDT had a shorter telomere length as reflected by the lower T/S ratio when compared with healthy controls. The telomere shortening was associated with the age and pre-transfusion Hb level. This finding suggested that patients with severe thalassemia exhibited accelerated telomere shortening. There was no statistically significant correlation between telomere length and other factors such as sex, reticulocyte count, ferritin, cardiac T2*, LIC, NTBI, and hemolysis parameters.

A recent study in patients with beta-thalassemia/HbE disease showed that accelerated telomere shortening was found only in patients who had severe clinical symptoms. Telomere length associated with aging was only found in patients with mild clinical symptoms but not in the groups with moderate and severe clinical symptoms. Reticulocyte count showed a correlation with telomere shortening. It was suggested that enhanced erythropoiesis in beta-thalassemia/HbE disease caused accelerated telomere shortening and demonstrated a disease-dependent trend that was not related to aging but is associated with reticulocytes [11]. This finding was in agreement with our study in that the more clinical severe the symptoms, the shorter of telomere length. The difference in our study was the identification of pre-transfusion Hb level as an indicator for disease severity, and not reticulocyte count. The explanation for this may be from the cross-sectional information as a spot reticulocyte count value may not represent the true reticulocyte count. In addition, some patients had other medical conditions that could influence reticulocyte count such as endocrinopathy and heart disease [16, 17].

Elevated body iron level leads to increased oxidative stress and long term exposure to oxidative stress leads to accelerated telomere shortening. Studies of hereditary hemochromatosis showed that elevated iron levels were associated with telomere shortening [18, 19]. However, we found no correlation between serum ferritin level and telomere length. This result was similar to the report in beta-thalassemia/Hb E disease [9], but differed from the findings in hereditary hemochromatosis [18, 19]. This may be explained by the different population in the study and the method of body iron measurement. However, in our study, telomere length in patients who had LIC > 15 mg/g had a tendency to be shorter than those in the group of low LIC but the findings were not statistically significant. Tissue iron may represent the body iron status of patients and is associated with accelerated shortening telomere length.

From our study, two patients who did not receive iron chelation due to severe adverse reactions to all chelation drugs had a shorter telomere length than other patients who received iron chelation. Patients who received deferoxamine had the longest telomere length. We hypothesized that the antioxidant effects of deferoxamine might be the protecting factor [20]. Other previous studies showed that an antioxidant may help to protect against telomere length shortening [21, 22]. However, this finding about iron chelators needs to be interpreted carefully as patients who needed combination iron chelators may have had higher iron burden and experienced more chronic oxidative stress. Further studies should be conducted to evaluate the impact of iron chelation on telomere length.

The limitation of this study was that the telomere length was measured only by the MMQPCR method, but not by the Southern blot method which was the gold standard. However, our previous study validated the MMQPCR method which was described by Cawthon et al. by comparing with the standard telomere length analysis. The results confirmed that the MMQPCR technique was a good method to measure telomere length. It would be interesting in the future to explore the telomere length in NTDT patients and compare with TDT patients and controls, as the disease pathophysiology is different and NTDT patients have a wide spectrum of disease severity.

## Conclusion

Our study showed that patients with TDT had shorter telomeres. Telomere shortening in TDT thalassemia patients was an age-dependent process and was associated with greater clinical severity. Iron chelation may have a role to control telomere shortening. Further studies are needed for drugs targeting telomeres, for example testosterone that has been used for telomere elongation in patients with telomere diseases [23, 24] and antioxidants that can protect telomeres from oxidative stress [21, 22] for their role in improving the treatment outcomes in patients with TDT.

## Data Availability

The data that support the findings of this study are available from the corresponding author, A.T., upon reasonable request. The data are not publicly available due to privacy or ethical restrictions.

## Abbreviations

TDT: Transfusion-dependent thalassemia
NTDT: Non-transfusion dependent thalassemia
PCR: Polymerase chain reaction
Hb: Hemoglobin
LFT: Liver function test
NTBI: Non-transferrin bound iron
T2*: MRI for cardiac T2 star
LIC: Liver iron concentration
